# Synchrony of change in depressive symptoms, health status, and quality of life in persons with clinical depression

**DOI:** 10.1186/1477-7525-4-27

**Published:** 2006-04-25

**Authors:** Paula H Diehr, Ann M Derleth, Stephen P McKenna, Mona L Martin, Donald M Bushnell, Gregory Simon, Donald L Patrick

**Affiliations:** 1 Department of Biostatistics F-670 HSB, Box 357232, University of Washington, Seattle, WA 98195-7232, USA; 2 Galen Research, Enterprise House, Manchester Science Park, Lloyd Street North, Manchester, M15 6SE, UK

## Abstract

**Background:**

Little is known about longitudinal associations among measures of depression, mental and physical health, and quality of life (QOL). We followed 982 clinically depressed persons to determine which measures changed and whether the change was synchronous with change in depressive symptoms.

**Methods:**

Data were from the Longitudinal Investigation of Depression Outcomes (LIDO). Depressive symptoms, physical and mental health, and quality of life were measured at baseline, 6 weeks, 3 months, and 9 months. Change in the measures was examined over time and for persons with different levels of change in depressive symptoms.

**Results:**

On average, all of the measures improved significantly over time, and most were synchronous with change in depressive symptoms. Measures of mental health changed the most, and physical health the least. The measures of change in QOL were intermediate. The 6-week change in QOL could be explained completely by change in depressive symptoms. The instruments varied in sensitivity to changes in depressive symptoms.

**Conclusion:**

In clinically depressed persons, measures of physical health, mental health, and quality of life showed consistent longitudinal associations with measures of depressive symptoms.

## Background

The constructs of depression, mental and physical health, and quality of life (QOL) are believed to be distinct but closely related. Depressed persons had worse health status and QOL than others in several cross-sectional comparisons [1-6]. Cross-sectional data cannot address whether changes over time in one construct are reflected in the other areas as well, which requires experimental or longitudinal data. Only a few studies in primary care and specialty samples have examined longitudinal associations between depression and measures of health status or disability. In a sample of high utilizers of general medical care, improvement in depression over 12 months was associated with significant reductions in both days of disability due to illness and in self-rated disability [7]. In a sample of primary care patients, long-term (1 to 3 year) improvement in depressive and anxiety symptoms was associated with significant improvement in interviewer-rated social disability [8]. In a sample of specialty care patients, month-to-month changes in severity of depression were associated with similar changes in interviewer-rated psychosocial health [9]. Persons with clinical depression experienced changes in both depression and quality of life over a 6-week period [10].

Here, we expand on these earlier findings by examining a wider range of health status and quality of life measures as well as examining synchrony of change over both short (6 weeks) and intermediate (9 month) time frames. We examine synchrony between the three constructs (depression, QOL, and health status) and agreement among the instruments used to operationalize the constructs. The primary goal of this paper is to determine whether mental health, physical health, and quality of life changed over time in persons who were clinically depressed at baseline, and to determine whether the change in depressive symptoms was synchronous with change in the other measures. The second goal is to compare the performance of the various instruments in a cohort of persons who were initially clinically depressed, to determine which instruments showed the most change, and to provide some information about the psychometric properties of each instrument.

## Methods

The overall aim of the Longitudinal Investigation of Depression Outcomes (LIDO) study was to characterize associations among quality of life (QOL), economic and depression outcomes, based on a multi-national observational study involving a prospective cohort of primary care patients with major depressive disorder [6]. LIDO investigators evaluated depressive symptoms, mental and physical health, and quality of life for 982 clinically depressed persons from 6 international research sites: Be'er Sheva, Israel;Barcelona, Spain; Porto Alegre, Brazil; Melbourne, Australia; St. Petersburg, Russia; and Seattle, WA, USA.

Patients making primary care visits were screened for initial eligibility, defined as a score of 16 or greater on the Center for Epidemiologic Studies- Depression scale (CESD) [11]. For patients meeting initial eligibility criteria, a baseline assessment was conducted, which included administration of a depression diagnostic instrument, the Composite International Diagnostic Interview (CIDI) [12,13]. All subjects who were diagnosed as clinically depressed by the CIDI (i.e., satisfied DSM-IV criterion A for major depressive episode [14]) were considered eligible, but those with a known organic or major psychiatric disorder (dementia, psychosis, bipolar disorder) were excluded. Patients who currently or in the previous three months were under treatment for depression were also excluded. The reference population is thus persons with untreated clinical depression being seen in primary care settings. Those who met the inclusion criteria and were willing to enroll in the study were followed for depression, health, and QOL at baseline, 6 weeks, 3 months, and nine months.

### Measures

Depressive symptoms were measured by the CESD scale [11], a 20-item scale designed to measure symptoms of depression in community populations. A higher value indicates worse depression and a cut point of 16 is generally used to distinguish individuals considered to be depressed [15]. Depression according to the DSM-IV Criterion A was assessed at baseline and 9 months using the CIDI [12].

Health status may be defined using the World Health Organization's definition of health as "a state of complete physical, mental, and social well-being, and not merely the absence of infirmity" [16]. Mental and physical health status were measured using the Short-Form 12 (SF12) [17-19] plus three additional questions about mental health which, combined with two questions in the SF-12, make up the Mental Health Subscale (MHI5) of the SF-36 [20]. For this paper we used: the physical and mental component scores (PCS and MCS); the MHI-5; and responses to three single questions "Have you accomplished less than you would like as a result of any emotional problems (such as feeling depressed or anxious)" (Do less:Emotional), "Have you accomplished less than you would like as a result of your physical health" (Do less:Physical), and the general health question "Is your health excellent, very good, good, fair or poor" EVGGFP). In all cases, higher values were coded to represent better health.

Because one goal of the over-all LIDO study was to compare Quality of Life (QOL) instruments, several of them were administered. These included the Quality of Life in Depression Scale (QLDS) [21,22] and the World Health Organization Quality of Life Brief Questionnaire (WHOQOL Bref) [23,24]. The QLDS, which employs the needs-based model of QOL, has 34 items, each requiring a "true/yes" or "false/no" response. The score is an index ranging from 0 to 34, with a higher score indicating worse QOL. The WHOQOL Bref adopts the following definition of health-related QOL: "the value assigned to duration of life as modified by the impairments, functional states, perceptions, and social opportunities that are influenced by disease, injury, treatment, or policy" [16]. It is a 26-item measure [23,24] that provides scores ranging from 4 to 20 for four separate domains (WQ:Physical; WQ:Psychological; social relationships WQ:Social; and WQ:Environment). We also included the single item "How would you rate your quality of life" (QOL:Rate). Higher scores indicate better quality of life.

### Analysis

This paper deals with the 982 persons (of the 1180 enrolled) who had a CIDI assessment at 9 months. In a few cases where data were missing at one time, but available at the prior and subsequent times, we substituted the average of the person's values before and after the missing observation, an approach that yielded reasonably unbiased estimates for the CESD and EVGGFP in a different study [25]. Responses to the EVGGFP question were transformed according to the probability the person will be in Excellent, Very Good or Good health one year in the future, to make the average value more interpretable [26].

To make it easier to compare the measures, we converted all of the scale values to z-scores, and changed the signs on the CESD and QLDS z-scores, so that a higher value would always represent better status. The z-scores were calculated from the means and standard deviations of all values across the 9 month period (shown in Table 1). If there is improvement over time, the early z-scores should tend to be negative, and the later ones should be positive.

**Table 1:** Descriptive Statistics of Measures (Original Scale) (N = 982 persons)

	**Grand Mean**	**Standard Deviation**	**Baseline**	**6 weeks**	**3 months**	**9 months**	**9 months minus baseline**
Age of participant	40.77	14.59					
% Female	70.98	45.39					
% Depressed at 9 Months^1^	35.64	47.90					
							
CESD^2 ^*	24.31	12.12	29.03	24.62	23.02	20.55	-8.48
MCS^3^	37.95	11.28	34.05	37.55	39.19	40.99	6.94
MHI5^3^	51.13	20.82	43.91	50.40	53.38	56.81	12.9
Do Less:Emotional	1.35	0.48	1.23	1.33	1.38	1.46	.23
							
PCS^3^	42.83	11.28	42.01	42.49	43.09	43.72	1.71
Do Less:Physical	1.40	.49	1.30	1.39	1.43	1.47	.17
EVGGFP ^3^	56.13	28.13	54.01	54.85	56.66	59.01	5.00
							
QLDS ^4 ^*	10.18	7.99	11.94	10.52	9.70	8.58	-3.36
QOL:Rate^5^	3.25	0.85	3.17	3.25	3.26	3.33	.16
WQ:Environment^5^	12.54	2.51	12.26	12.39	12.59	12.92	.66
WQ:Physical^5^	12.87	3.00	12.16	12.75	13.05	13.52	1.36
WQ:Psychological^5^	12.04	2.69	11.41	11.91	12.23	12.60	1.19
WQ:Social Relationships^5^	12.47	3926	11.95	12.34	12.57	13.02	1.07

* High values represent worse status

1. Based on Comprehensive International Diagnostic Interview, Version 2.1, Section E (Weiller et al. 1994)

2. CESD. Center for Epidemiologic Studies-Depression. (Radloff 1977). Higher scores indicate worse depression.

3. Based on the Medical Outcome Study Short-Form 12 Questionnaire (SF-12) (Tarlov et al 1989, Steward & Ware, 1992): MCS = Mental Component Scale; MHI-5: 5-item summative mental health scale using 2 questions from SF-12 and 3 additional items; Do less:Emotional 1=yes, 2=no; PCS = Physical Component Scale; Do Less:Physical 1=yes, 2=no; EVGGFP = General health question transformed to probability of being in Excellent, Very Good or Good health at 1 year: Excellent = 95, Very Good = 90, Good = 80, Fair = 30 or Poor = 15. (Diehr, 2001)

4. QLDS Quality of Life – Depression Scale (McKenna & Hunt 1992, Hunt & McKenna 1992) Higher scores indicate worse quality of life.

5. WHOQOL-Bref (WHOQOL Group, 1995, 1996). QOL:Rate is a 5-point scale rating quality of life. All other scores on 0–100 scale: Overall Quality of Life (QOL:Rate), and Environment, Physical, Psychological,, Social Relationships domains.

We examined change in each variable over time. We also compared change among subsets of patients defined by either their change in the CESD from baseline to 6 weeks, or by their CIDI at 9 months. To determine whether there were changes in the other measures independent of the changes in the CESD, the 6-week change in each measure was regressed on the 6-week change in the CESD scores and examined for the statistical significance of the constant term, which is an estimate of the change in the measure of interest for persons who had no change in the CESD. A second set of regressions added CESD squared.

When the dependent variable is a measure of change, subjects are essentially their own controls. To determine whether personal and site characteristics interacted with change in important ways, in some analyses we adjusted the change measures for age, gender, education and site and also examined change separately by site.

Because the research question is complex and 13 different variables are examined, we chose to make the analysis simple, primarily based on descriptive statistics and simple regression analysis.

## Results

Of the 1180 clinically depressed persons enrolled, 982 had a valid CIDI value at 9 months. Persons without a valid CIDI were similar in age and sex to those with a CIDI, but had significantly better baseline health. For the 982 persons studied here, mean age was 41 (range 17–76) and 71% were women. Fewer than one percent had missing data. Table 1 shows the grand mean and standard deviation for each variable, for all times combined. Recall that higher scores on the CESD and the QLDS domains indicate worse depression or health, respectively (denoted by an asterisk in the table), while higher scores on the other measures indicate better status. Table 1 also shows the mean value of each study variable at the four time periods. For example, the grand mean for CESD was 24.31, and the mean score decreased from 29.03 at baseline to 20.55 at 9 months, or a decrease of 8.48 CESD points. Thus, although there was improvement, the average level of depressive symptoms was still high 9 months after baseline. All of the measures showed improvement over time. The standard deviations of change scores from screener to baseline and from baseline to 6 weeks, 3 months, and 9 months are in Appendix Table 1. These may be useful for estimating the necessary sample size for future studies of clinically depressed patients.

Table 2 shows the average z-score for each variable, at each time point. The z scores represent differences from the mean in standard deviation units,(i.e., the observed value minus the grand mean divided by the overall standard deviation) using the values reported in Table 1. The signs were reversed for the CESD and QLDS, so that a higher value indicates better status for all variables. On average, all variables were below the grand mean at baseline (the z-score has a negative sign), and all but CESD and QOL:Rate were still negative at 6 weeks. All the variables were above the mean at 3 months and 9 months. Thus, all variables showed monotone improvement over time (baseline < 6 weeks < 3 months < 9 months). The largest change over time was for the CESD, which moved from .36 standard deviations below the mean at baseline to .33 standard deviations above at 9 months, or an improvement of .69 standard deviations. The mental health variables all showed large changes, the physical health variables a smaller amount of change, and the QOL variables were intermediate.

**Table 2:** Mean Z-Scores^1 ^over time (N = 982)

	**baseline**	**6 weeks**	**3 months**	**9 months**	**9 months minus baseline**
CESD (reversed)^2^	-.36	.00	.13	.33	.69
					
MCS	-.33	-.02	.13	.29	.62
MHI5	-.35	-.03	.11	.27	.62
Do Less: Emotional	-.26	-.05	.06	.24	.50
					
PCS	-.07	-.03	.02	.08	.15
Do Less:Physical	-.18	-.01	.07	.16	.34
EVGGFP	-.07	-.04	.02	.10	.17
					
QLDS (reversed)^2^	-.19	-.02	.09	.23	.42
QOL:Rate	-.09	.01	.02	.10	.19
WQ:Environment	-.10	-.05	.03	.16	.26
WQ:Physical	-.23	-.03	.07	.23	.46
WQ:Psychological	-.22	-.03	.09	.22	.44
WQ:Social Relationships	-.14	-.02	.04	.17	.31

1 The z scores represent differences in standard deviation units, (i.e., the mean at each time point minus the grand mean of all the observations divided by the overall standard deviation) using the values reported in Table 1, with the signs reversed for the CESD, and QLDS, so that a higher value indicates better status for all variables

2 Sign reversed so that higher value indicated better status for all measures

We next examined whether changes in the CESD were mirrored by changes in the other variables. The subjects were divided into four (approximately) equal groups on the basis of their 6-week change in the CESD. Table 3 shows the mean change (in the z-scores) between 0 and 6 weeks. A positive change represents improvement. The first column in Table 3 has information for those whose CESD improved by 10 or more points in 6 weeks (chosen because about 25% of the subjects improved 10 or more points). The mean improvement for the CESD for this group was of course high, because the groups were defined by CESD change; persons averaged an improvement of 1.50 standard deviations on this measure. All of the other variables in column 1 also had positive 6-week change, but the amount of change was smaller. This positive change was statistically significant (denoted by the "+" sign following the values in this column) for all but the PCS. (Change on the original scale can be calculated by multiplying the change in z-score by the appropriate standard deviation in Table 1). The fourth column of Table 3 shows the means for persons whose CESD worsened by 2 or more points in the first 6 weeks. Their CESD dropped an average of .62 standard deviations. Most of the other variables also had negative change. The only exception was PCS, which did not change. The "-" signs following numbers in this column denote variables with significant negative change. Most of the mental health and QOL measures showed a statistically significant decrease in column 4, but none of the physical health measures changed significantly.

**Table 3:** Average 6 Week Change (in Z Scores) by Quartiles of 6-Wk Change in CESD

**Quartile of Change in the CESD**					
**6-week Change in z-scores**	**Improved >10 on CESD**	**Improved 3–10**	**No Change -2 to +3**	**Worse -2 or less**	**Mean for Improved minus Mean for Worse**
N	243	241	244	254	243
					
CESD (reversed)	1.50+	.55+	.06+	-.62-	2.12
MCS	.93+	.39+	.13+	-.19-	1.12
MHI5	1.03+	.38+	.13+	-.26-	1.29
Do Less:Emotional	.53+	.27+	.16+	-.11	.64
					
PCS	.08	.02	.05	0	.08
Do Less: Physical	.39+	.22+	.13	-.04	.43
EVGGFP	.21+	-.02	-.04	-.04	.17
					
QLDS (reversed)	.71+	.25+	.05	-.27-	.98
QOL:Rate	.42+	.20+	.06	-.28-	.70
WQ:Environment	.29+	.13+	.01	-.21-	.50
WQ:Physical	.64+	.28+	.07	-.18-	.82
WQ:Psychological	.78+	.24+	.02	-.28-	1.06
WQ:Social Relationships	.54+	.13+	.06	-.27-	.81

+ following the entry: Significantly greater than zero (significant improvement).

- following the entry: Significantly less than zero (significant decline).

At the end of the study all persons were assessed for clinical depression using the CIDI interview. Table 4 shows the mean 9-month change in each variable as a function of clinical depression status at 9 months. All of the measures improved significantly over time for those who were not clinically depressed at 9 months. The group with persistent depression did not change significantly on any of the measures.

**Table 4:** Mean Change in 9 months (Z-Scores) by 9-month Depression Status (from the CIDI)

	**Not Clinically Depressed**	**Clinically Depressed**	**Not Depressed minus Depressed**
N	631	350	
CESD (reversed)	1.05+	.07	.98
			
MCS	.95+	.00	.95
MHI5	.96+	.01	.95
Do Less:Emotional	.75+	.05	.70
			
PCS	.21+	.08	.13
Do Less: Physical	.48+	.10	.38
EVGGFP	.28+	-.01	.29
			
QLDS (reversed)	.63+	.04	.59
QOL:Rate	.33+	-.06	.39
WQ:Environment	.41+	.01	.40
WQ:Physical	.68+	.06	.62
WQ:Psychological	.72+	-.06	.78
WQ:Social Relationships	.47+	.03	.44

+ following the entry: Significant improvement (greater than 0).

The data were collected in six international sites, and there were significant differences among sites in the baseline levels of the measures [6]. In spite of this, the age-sex-education-site-adjusted changes were nearly identical to the unadjusted results. Further, graphs of changes over time were very similar across sites. For these reasons, as well as for space constraints, results are not presented separately by site or by other covariates.

We investigated whether there were changes in each measure, independent of changes in the CESD, by regressing 6-week change for each instrument on 6-week change in the CESD; for example, we regressed change in the QLDS on change in the CESD. We then tested whether the constant term in the regression was significantly different from zero. That is, did the average person with no change on the CESD have significant change in the variable of interest (QLDS in the example)? This is conceptually similar to what is shown in column 3 of Table 3. There was significant improvement in the first 6 weeks, even after controlling for change in the CESD, for MCS, MHI5, Do less:Emotional, Do less: Physical, and for WQ:Physical. There was not significant change in PCS, EVGGFP or in any QOL measure but WQ:Physical, which became non-significant if CESD squared was added to the regression. In a final analysis of the baseline data only, we regressed each QOL measure on CESD, and then used stepwise regression to select additional significant predictors from among age, sex, education, and the three physical health measures. Two or more of these candidate variables entered in every regression, and will be addressed below.

## Discussion

The primary goal of this paper was to determine whether mental health, physical health, and quality of life changed over time in persons who were clinically depressed at baseline, and to determine whether the change was synchronous with change in depressive symptoms. We also wanted to know whether change in depression could explain the changes in the other dimensions. The final goal was to compare the performance of the various measures of each construct.

### Changes in mental health, physical health, and quality of life

All of the measures improved significantly over time, with the measures of mental health improving the most, physical health the least, and QOL showing intermediate change. The mean changed monotonically with time (Table 1 and Table 2). The change was synchronous with 6-week change in the CESD (Table 3), in that persons who improved the most on the CESD also showed the most improvement in the other measures (not significant for the PCS). Similarly, those whose CESD worsened in the 6 weeks after baseline tended to worsen on mental health and QOL as well. Physical health did not change significantly in that group. Consistent with previous studies [7-10] we found strong evidence for synchrony of change in depressive symptoms and measures of either health status or quality of life. Similar results were seen for both short (6-week) and intermediate (9-month) time frames.

Since all persons were initially depressed and most improved, it is likely that improvement in the underlying depression caused the changes in the other dimensions, and that this improvement had the most impact on mental health, the least on physical health, and intermediate impact on quality of life. However, there are other possible interpretations. It may instead be that the underlying physical problem that brought the subjects to the doctor was resolved, having a positive effect on all three constructs. We could not address this possibility because no data were available on the subjects' reason for their initial primary care visit.

These findings may indicate that the constructs of mental health, physical health, and quality of life are highly related, both cross-sectionally and over time, with mental health and quality of life more similar to each other than to physical health. Another possibility, however, is that it is the imperfect measures of mental health, physical health, and quality of life that are highly correlated, in part because they contain similar items. McKenna has classified the CESD, MHI5, SF12, WHOQOL, and QLDS instruments as to whether they measure impairment, disability, handicap, or quality of life. Most of the instruments include items about impairment and disability, but only the WHOQOL-environmental measures handicap, and only the QLDS and the WHOQOL-psychological measure quality of life [27]. Changes in QOL would be expected to be less than changes in symptomatology following Brenner's first principle: The greater the distance between disease and outcome measure, the weaker the association between them will be [28]. In the present case CESD is close to the severity of depression as it is intended to measure symptomatology. The QLDS is further from the disease as it is affected by other influences on the patient such as personality, economic resources and social support.

We found that, after controlling for change in the CESD, there remained significant additional change in the measures of mental and physical health, but not in QOL. This suggests that observed changes in the measures of mental and physical health reflect more than just changes in the depressive symptoms as measured by the CESD. Since change in CESD could completely explain changes in the QOL measures, it is possible that change in QOL measures only change in depression. Alternatively, this finding could simply mean that the components of QOL that were unrelated to depression did not change, on average, during the study period. Although a full study of the components of QOL is beyond the scope of this paper, we did test whether, in the baseline data, a patient's age, sex, education, or physical health was related to each QOL measure after controlling for CESD, and for each QOL variable two or more additional variables were significant predictors. Thus, QOL is not simply a measure of depression. However, in this population, there was considerable change over time in depression but little or no change in age, sex, education, or physical health, and all of the change in QOL could be explained by the change in depressive symptoms. If this finding holds true in other studies, it may not be necessary to measure QOL changes in settings similar to this one.

### Comparison of measures

We next discuss the individual measures within each larger type, by their sensitivity to differences or changes in depressive symptoms. The measure of sensitivity could be defined as either the mean change for those whose 6-week CESD improved more than 10 points minus the change for those whose CESD worsened by 2 or more points (Table 3), or as the mean change for those who were not clinically depressed at nine months (Table 4). As results using these definitions agreed substantially, we refer simply to the sensitivity of a measure. Formal calculation of the effect size and responsiveness of each measure in this population may be performed using data in Appendix 1 [29].

### Mental health

All of the mental health measures were sensitive to change in depressive symptoms, which is not surprising, since they include many measures of depressive symptoms. The sensitivity of the MCS and MHI5 was similar. The single item Do less:Emotional, "accomplished less than you would like for emotional reasons" was less sensitive. The finding that change in the CESD did not completely predict the change in the other measures suggests that they have some different content, which may be important in some settings.

### Physical health

The physical health items were less sensitive to depression changes than were the mental health or quality of life measures. This may suggest that the relation of change in physical health to change in depressive symptoms is not strong. However, this comparison may be biased by regression to the mean. Subjects were recruited in primary care clinics, where they may have presented for acute illness, and their natural improvement in physical health may have masked the changes related to their depression state. However, when we adjusted the change in PCS for the baseline value of PCS, the estimated changes were no different, suggesting that this regression to the mean was not a major factor in the lack of relationship.

The PCS was the least sensitive physical health measure. The PCS has the feature that if a person has no change on 7 of the SF-36 subscales but improves on the mental health subscale, his PCS will actually decrease (and similarly, improvement in physical health can lower the MCS value) [30,31]. Here, persons whose mental health improved over time did not show much improvement in the PCS, even though the Do less:Physical measure improved substantially. Thus, the finding of lower sensitivity of the PCS to depression may be in part an artifact, although others have argued that the artifact is not a problem [32].

### Quality of Life

The sensitivity of the QOL measures to depressive symptoms was intermediate between the mental health and the physical health measures. All of the QOL measures were sensitive to depression, but there was wide variation. The WQ:Psychological and the QLDS were designed specifically to measure the mental health aspects of QOL. As expected, these two measures were the most sensitive to depressive symptoms. Surprisingly, however, the WQ:Physical, designed to measure physical QOL, also showed similar change to the QOL measures. This finding demonstrates the importance of examining the content of the instruments. The WQ:Physical, for example, includes items about sleep problems, which are also emphasized in the CIDI and CESD, and which may explain why it was so sensitive to depressive symptoms. (This many imply that WQ:Physical is not measuring what is intended) As mentioned above, only the QLDS and WQ:Psychological were felt to measure the construct of quality of life [27].

### Single items

The single-item measures were sensitive to changes in depressive symptoms, although usually less sensitive than the multi-item measures. For large studies in which there is concern about respondent burden, such measures might be a "cost-effective" alternative to longer instruments. For example, under certain assumptions Do less:Emotional is cost effective relative to the MHI5 [29]. Similarly, the sensitivity of the MHI5 was as good as that of the MCS. The face validity of a measure consisting of only one or a few items, however, may not be satisfactory for many purposes. Others have found single-items to be useful in clinical situations. [33,34]

### Implications for clinicians, QOL research, and choosing study instruments

These findings have implications for clinicians, for QOL researchers, and for investigators choosing an instrument. Clinicians may expect measured QOL (and, to a lesser extent, physical health) to improve in synchrony with improvements in depressive symptoms. If measuring changes in QOL does not provide additional information in studies of clinically depressed patients, it may not be necessary to measure QOL after baseline. (However, it would then not be possible to state with certainty that QOL had improved). We may not need more research to answer the question "Does QOL improve when depression improves?" But it may be worth measuring changes in QOL if the goal is to assess the public health importance of improvement in depression or to compare the effect of depression on QOL to the effects of other health conditions.

In choosing an instrument for a new study, an important task for the investigator is to examine the domains covered by the instruments, to ensure that they are likely to be affected by the intervention of interest. Other important issues in instrument selection are that any scale selected must be shown to be unidimensional, allowing scores to be added and to have construct validity. Among acceptable instruments, the investigator might then choose the one that is the most reliable, that imposes the least subject burden, or that permits the study to be performed with the smallest sample size, depending on the needs of the study. The information about the sensitivity of the various instruments to changes in depressive symptoms may be useful, in that the most sensitive instrument will permit studies with the smallest sample sizes. Additional information is available in Appendix 1.

### Limitations

Study subjects were recruited in primary care clinics, and were probably sicker than the general public at baseline. These results may not generalize to persons with less severe depression. Persons who refused or had missing data at 9 months were not included in this analysis. The LIDO study included only self-reported measures of health status and quality of life. Some have questioned whether depressed mood leads to biased self-reports of health and social adjustment [35,36]. Previous studies, however, have observed similar longitudinal associations, whether health was measured by self-report [7] or interviewer ratings [8,9]. Furthermore, a similar synchrony of change has been observed for depression and number of days missed from work due to illness, a measure presumably less subject to bias [37]. Although data were collected in six sites, there were similar results when patient and site characteristics were included in the analysis. Only simple analytic methods were employed. Further insights might have arisen from such approaches as structural equation modeling. We could not determine the causality of the synchrony. We did not have information on clinical status at baseline or over time, which would be needed to rule out the possibility that the resolution of some acute health problem caused the synchronous improvement over time in the three domains.

## Conclusion

All measures improved significantly, in synchrony with changes in clinical depression. Measures of depressive symptoms and mental health showed the greatest change, followed by quality of life, and then by physical health. The single items performed fairly well, and may be a useful alternative in some large studies. These findings may be useful for future researchers who need to choose among instruments. Additional research in these areas should further examine causality by following clinical changes in the patients. Further studies of the importance of changes in QOL in subjects with clinical depression are also needed.

## Competing interests

The author(s) declare that they have no competing interests.

## Authors' contributions

All authors contributed to the data collection, design, analysis, and writing of this manuscript.

**Table 5:** Appendix Table 1: Standard Deviation of Change from Baseline (original scale)

	Screener to Baseline *	**Baseline to 6 weeks**	**Baseline to 3 months**	**Baseline to 9 months**
CESD	8.75	10.48	11.65	13.67
				
MCS	9.49	10.83	11.77	13.06
MHI5	17.18	19.08	20.55	23.45
Do Less:Emotional	0.47	0.50	0.54	0.56
				
PCS	7.74	8.02	8.77	9.69
Do Less:Physical	0.50	0.52	0.53	0.55
EVGGFP	0.68	0.67	0.73	0.81
				
QLDS		6.11	6.41	7.40
QOL:Rate		0.81	0.83	0.91
WQ:Environment		1.78	2.00	2.15
WQ:Physical		2.25	2.55	2.86
WQ:Psychological		2.17	2.42	2.78
WQ:Social Relationships		2.92	3.16	3.38

* The standard deviation of the difference between the screening value and the baseline value 14 days later is for persons whose screening value was 16 or higher. The last 6 instruments were not used in the screening population.

The information in Appendix Table 1 should be useful to others in planning future studies of clinically depressed persons. For example, a study might be designed to affect the change in QOL over 3 months as measured by the QLDS. The 3-month change-score had standard deviation 6.41. To detect a difference of, say, 2 points between a treatment and control group with 80% power, the necessary sample size is (1.96+.84)^2 ^* 2 * 6.41^2 ^/2^2 ^= 161 persons per treatment group. The effect size or responsiveness of an instrument can be calculated from this information as well [29]. Finally the test-retest reliability of the instrument for a population with one CESD score above 16 and a positive CIDI assessment may be approximated from the screener-to-baseline information.
